# The cost of healthy versus current diets in the Netherlands for households with a low, middle and high education

**DOI:** 10.1016/j.ssmph.2022.101296

**Published:** 2022-11-25

**Authors:** Jody C. Hoenink, Wilma Waterlander, Stefanie Vandevijvere, Joline W.J. Beulens, Joreintje D. Mackenbach

**Affiliations:** a Amsterdam UMC, Vrije Universiteit Amsterdam, Department of Epidemiology and Data Science, Amsterdam Public Health Research Institute, De Boelelaan, 1117, Amsterdam, the Netherlands; b Upstream Team, Amsterdam UMC, the Netherlands; c Amsterdam UMC, University of Amsterdam, Department of Public and Occupational Health, Amsterdam Public Health Research Institute, Meibergdreef 9, Amsterdam, the Netherlands; d Scientific Institute of Public Health (Sciensano), Department of Epidemiology and Public Health, Brussels, Belgium; e Julius Center for Health Sciences and Primary Care, University Medical Center Utrecht, Utrecht University, Utrecht, the Netherlands

**Keywords:** Food prices, Socioeconomic inequalities, SES, Socio-economic status, INFORMAS, Modelling

## Abstract

The cost of food is an important driver of food choice and most evidence suggests that healthier diets are more costly than less healthy diets. However, current attempts to model the cost of healthy and current diets do not take into account the variation in diets or food prices. We calculated the differential cost between healthy and current diets for households with a low, medium and high education in the Netherlands using the DIETCOST program. The DIETCOST program accounts for variations in dietary patterns and allows for the calculation of the distribution of the cost of bi-weekly healthy and current household diets. Data from the Dutch National Food Consumption Survey 2012–2016 was used to construct commonly consumed food lists for the population as a whole and for households with a low, medium and high education and linked to a local food price database. The average cost of current household diets was €211/fortnight (SD 8.9) and the healthy household diet was on average €50 (24%) more expensive. For households with a low, medium and high education, healthy diets were on average 10% (€17), 26% (€50) and 36% (€72) more expensive compared to current diets, respectively. All healthy diets could be classified as affordable (i.e. requiring less than 30% of the average disposable income) as diets required around 20% of the income. To conclude, while healthy diets were found to be affordable, we found that these were more expensive than current diets, especially for those with a higher educational level. This suggests that individuals will need to spend more money on food if they aim to adhere to dietary guidelines under the assumption that they will minimally adjust their diet. Bridging the gap between the cost of healthy and less healthy foods could be an important strategy for improving population diets.

## Highlights

• Price of healthy and current Dutch diets were calculated using the DIETCOST program.
• DIETCOST program accounts for variations in dietary patterns.
• Cost of healthy fortnightly diet was €50 (24%) more expensive than current diet.
• Difference in cost increases by increasing household educational level.

## 1 Introduction

Although unhealthy diets are an important risk factor for non-communicable diseases (Habib & Saha, 2010), population diets in general do not meet recommended dietary guidelines for healthy eating. The Dutch guidelines state that adults should consume at least 200 g of fruit and 250 g of vegetables per day, which is achieved by only 13% and 16% of adults respectively (RIVM, 2021). When stratifying the percentage of adults who adhere to the fruit and vegetable guidelines by educational level, only 10% and 8% of adults with a lower educational level adhere to these guidelines compared to 17% and 24% of adults with a higher educational level (RIVM, 2021).

The cost of food is an important driver of food choice (Glanz et al., 1998) and most evidence suggests that healthier diets are more costly than less healthy diets (Rao et al., 2013). Findings from a review suggest that healthy diets are $1.50/day more expensive than current diets (Rao et al., 2013). However, current attempts to model the cost of healthy and current diets have limitations, including not accounting for the variation in diets or food prices. Therefore, the International Network for Food and Obesity/NCDs Research, Monitoring and Action Support (INFORMAS) developed a software program (DIETCOST) that allows researchers to model the differential in the cost distribution between healthy and current household diets. The program specifies constraints on nutrient and food intakes for healthy and current diets, and allows varied serving sizes of commonly consumed food items, leading to a range of household meal plans.

Because population diets, dietary guidelines and food prices vary from country to country, it is important to investigate the cost differential between healthy and current diets in multiple settings. For example, white meat is comparatively cheap in Australasia compared to Europe (Headey & Alderman, 2019). In Australia basic healthy foods are exempt from Goods and Services Taxes (GST) (Lee et al., 2020), while in the Netherlands the GST recently increased from 6% to 9% (NEA, 2021). Differences in price and taxation may in turn partly explain the findings from an Australian study suggesting that – contrary to most studies (Rao et al., 2013) – healthy diets are less expensive than current diets (Lee et al., 2020). Currently, the minimum cost of a healthy food basket in the Netherlands for a four-person household is €15.91/day (NIBUD, 2020). However, it is unclear what the cost differential is between healthy and current diets. Previous Dutch studies showed that higher dietary cost was associated with better dietary quality (Hoenink et al., 2020) and that higher energy dense diets were associated with lower diet costs (Waterlander et al., 2010). We therefore expect that healthier diets in the Netherlands are more expensive than current habitual diets (i.e. less healthy diets as current diets do not meet the Dutch dietary guidelines (van Rossum et al., 2020)).

It is also important to investigate the cost differential for different subpopulations. A review of the literature on diet quality and its socio-economic gradient found that high cost of healthy diets poses an especially important barrier to consume a healthy diet for households with a low socioeconomic position (SEP) (Darmon & Drewnowski, 2008). Although total spending on current diets is roughly equal between SEP groups (around 10–12% of disposable income (CBS, 2016)), households with the lowest SEP are most vulnerable to increased expenditure due to their limited food budget. Indeed, a recent Australian study found that both healthy and current diets were considered least affordable for the most disadvantaged households (Lee et al., 2020). By creating a list of commonly consumed foods for specific subpopulations in the DIETCOST program, the cost differential between healthy and current diets can be modelled for different subpopulations.

The current study aimed to calculate the differential in the cost and affordability between healthy and current diets for an average household as well as for households with a low, medium and high education (as a proxy for SEP) in the Netherlands using the DIETCOST program.

## 2 Methods

This study used the DIETCOST microsimulation model to estimate the cost of current and healthy diets of average Dutch household members as well as households with a low, medium and high education. The reference household for which the cost was calculated comprised of a 45-year-old man, a 45-year-old women, a 14-year-old boy and a 7-year-old girl. This modelling study was based on national population-level food consumption data and averages, which did not require the involvement of members of the public in the study conception, design, data analysis or reporting.

### 2.1 DIETCOST program

A comprehensive description of the algorithm and the use of the DIETCOST program can be found elsewhere (Mackay et al., 2017). Using a list of food items commonly consumed by the four household members, the DIETCOST model simulates multiple individuals’ fortnightly diets/meal plans (i.e. food items that are commonly consumed at breakfast, lunch, dinner and/or as a snack) that meet a pre-specified range of nutrient targets. Variations in the diets are created by setting nutritional constraints for healthy and current diets within which the serving sizes of food items in the healthy and current food baskets may vary. DIETCOST runs on input files including a list of foods commonly consumed by the population, nutrient targets and minimum/maximum constraints for certain food groups and nutrients part of healthy and current diets, healthy and current diet baskets, food composition data and food price data. The inputs for the DIETCOST program are further described below. A dataset example used for the total population can be found in Appendix A.

**Fig. 1:**
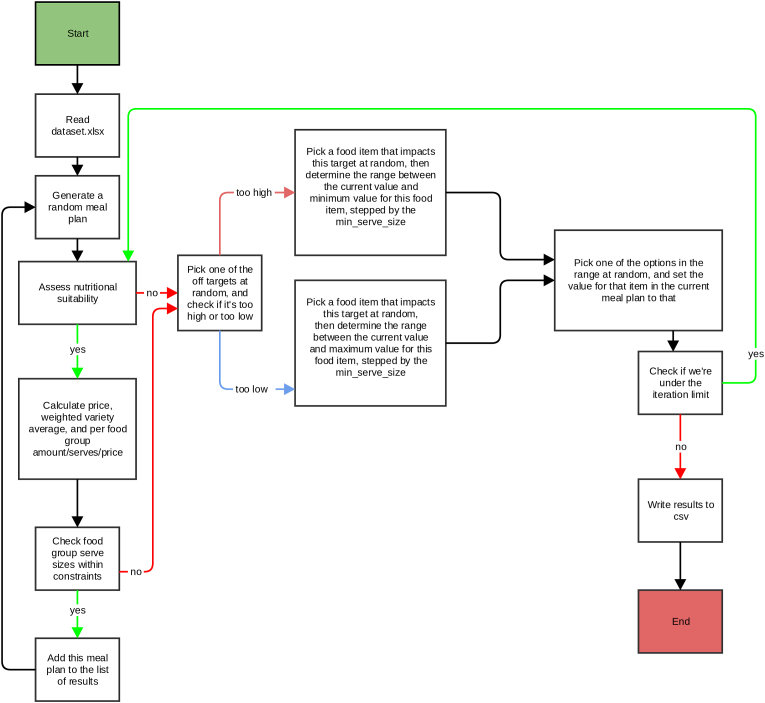
DIETCOST model algorithm Reproduced with permission from Vandevijvere S et al. Vandevijvere S, Young N, Mackay S, Swinburn B, Gahegan M. Modelling the cost differential between healthy and current diets: the New Zealand case study. International Journal of Behavioral Nutrition and Physical Activity. 2018 Dec;15(1):1–0*.*

Fig. 1 provides a flowchart of the DIETCOST program. If a meal plan meets the pre-specified nutrient targets, it is added to the list of meal plans. If the meal plan does not meet the nutrient targets, the DIETCOST algorithm will adjust the meal plan by randomly raising/lowering the serving size of some food item between the minimum and maximum serving size for that food item until the nutrient constraints are met. The output of the DIETCOST program includes a number of meal plans and its associated cost for each individual household member. The cost of each meal plan for each household member is then combined to calculate the distribution of the cost of a fortnightly current diet of an average Dutch household. This process is then repeated for healthy diets, including a different list of food items and a different pre-specified range of nutrient targets. This is done separately for the average Dutch household and the households with a low, medium and high educational levels.

### 2.2 List of commonly consumed foods

In accordance with the DIETCOST program manual, we constructed a list of commonly consumed foods for each household (i.e. the average Dutch household and households with a low, medium and high education) (Mackay et al., 2017). The commonly consumed food lists were derived using data from the Dutch National Food Consumption Survey (DNFCS 2012–2016). This survey consists of two 24-h dietary recalls on two random non-consecutive days. For children, the 24-h recall was combined with a food diary completed by the child's caretaker. Also, socio-demographic information was collected via questionnaires. DNFCS 2012–2016 study participants are representative for the Dutch population regarding their age, gender, region, degree of urbanisation and educational level (van Rossum et al., 2020). Educational level was measured by participants' highest completed educational level or, in case of participants aged under 19, that of the head of household. The response rate of the DNFCS 2012–2016 was approximately 65% (n = 4313). For this study, educational level was categorised into low (primary education, lower vocational education, advanced elementary education), medium (intermediate vocational education, higher secondary education) and high (higher vocational education and university) education.

The DNFCS 2012–2016 included 1854 unique food items and 18 distinguished food groups. All food items have a unique code that describes the food composition of that particular item. This Dutch Food Composition code (NEVO) was used to link nutrient information and whether the food is included in the Dutch dietary guidelines. Provided that no guidance about the construction of the commonly consumed food list was available, we decided to calculate the mean frequency food items were consumed by week for each of the four household members and used this as the cut-off. All food items consumed above this cut-off (a mean consumption of 13 for girls, boys and women, and 15 for men) were considered to be frequently consumed. We chose not to calculate this cut-off separately for each food group as using the cut-off across the entire diet gives a better representation of the average Dutch diet. If the food item was consumed more than the mean intake only for children or only for adults, this food was considered a commonly consumed food for either children or adults. Commonly consumed foods were found across a range of food groups, except for legumes, nuts and fish. Therefore, we selected additional healthy and less healthy foods within these food groups that were consumed just below the mean threshold. Overall, n = 200 food items were determined to be commonly consumed foods (Appendix A).

DNFCS data was then used to construct a list of food items commonly consumed by individuals with a low, medium and high education with education level-specific cut-offs, resulting in three separate datasets. For all three education categories, additional healthy food items needed to be included in the commonly consumed food list in the categories fruit, vegetables, grains, dairy and protein foods given the small amount of commonly consumed healthy food items within these categories. The largest difference between the datasets included the number of food items for the different households (in other words the level of variety); in total, 103, 137 and 149 food items were included in the commonly consumed food list of households with a low, medium and high education, respectively.

### 2.3 Nutrient composition

Next to a list of commonly consumed foods, information on the nutrient composition for each commonly consumed food was required. According to the DIETCOST manual, information on energy, fat, carbohydrates, sugars, fibre, protein and sodium is required, which was extracted from the NEVO database (RIVM, 2020a).

### 2.4 Nutrient targets and constraints

We set nutrient targets including minimum and maximum amounts (constraints) of consumption for food groups and macronutrients in the dataset file required for the DIETCOST program. These inputs were used to construct a variety of meal plans for which nutrient composition and food groups’ consumption for each meal plan fell within a set of (realistic) constraints. The DIETCOST program uses these constraints to then develop a number of realistic meal plans. The nutrient targets and constraints differed for healthy and current diets.

Input for the nutrition targets and constraints of current diets separately for the four household members were derived from a previously published DNFCS 2012–2016 report (van Rossum et al., 2020). Constraints for the macronutrients fat, saturated fat, carbohydrates, total sugars, fibre, protein and sodium were collected, including the amount of fruit, starchy vegetables, vegetables, dairy, grains and proteins consumed (Mackay et al., 2017). The nutrient constraints were then calculated by taking the average intake in grams of macronutrients and food groups ±30%, except for energy (which was equal to the mean intake ± 5%). For current diets, the nutrition targets for the DIETCOST model for individuals with a low, medium and high education were derived from the Dutch National Institute for Public Health and the Environment website (RIVM, 2020b). Unlike the report, the website reports the DNFCS 2012–2016 results stratified by age, sex and educational level, but only by two age categories (children and adults).

Information on the nutrient targets and constraints for a healthy diet were based on the Dutch dietary guidelines (Brink et al., 2018). These guidelines provide recommendations on the ideal minimum and/or maximum intake of food groups and nutrients stratified by sex and age. While the nutrient targets were completely based on the Dutch dietary guidelines, the nutrient constraints were based on a combination of the Dutch dietary guidelines and the World Health Organization population nutrient intake goals as an ideal range for macronutrients is not reported in the Dutch dietary guidelines (WHO). The nutrient targets and constraints for a healthy diet are equal for the overall population and the households with a low, medium and high education. Table 1 displays the nutrient and food group targets and constraints for the average Dutch household.

**Table 1:** Nutrient and food group input assumptions for the average Dutch household.

Nutrient/food group	Healthy diet guidelines (min-max)^a^				Current diet (min-max)^b^			
	Girls	Boys	Women	Men	Girls	Boys	Women	Men
Energy MJ	4.4–4.9	8.9–9.8	6.9–7.7	8.3–9.2	6.5–7.1	9.6–10.6	8.5–9.4	10.8–12.0
Fat in % energy	20–35	20–35	20–35	20–35	23–39	25–42	23–39	26–43
Saturated fat in % energy	0–10	0–10	0–10	0–10	9–15	9–15	8–14	9–15
Protein in % energy	15–25	15–25	15–25	15–25	9–16	10–17	10–17	11–18
Carbohydrates in % energy	45–65	45–65	45–65	45–65	39–66	36–61	30–50	31–52
Fibre in grams	18-max	36-max	30-max	35-max	10–19	14–26	13–24	17–32
Sodium in milligrams	192–962	403–2015	299–1493	383–1914	1293–2401	2145–3983	1891–3511	2637–4897
Red meat in grams	0–25	0–50	0–50	0–50	NA	NA	NA	NA
Dairy in servings	2.0-max	3.0-max	2.0-max	2.0-max	1.5–2.9	1.6–3.0	1.4–2.6	1.6–2.9
Vegetables in servings	1.0-max	2.0-max	2.0-max	2.0-max	0.7–1.2	0.9–1.8	1.3–2.3	1.3–2.4
Fruit in servings	2.0-max	2.0-max	2.0-max	2.0-max	0.7–1.4	0.5–1.0	0.7–1.2	0.5–1.0

a Data based on a combination of the Dutch dietary guidelines and the World Health Organization population nutrient intake goals. The nutrient targets and constraints for a healthy diet are equal for the overall population and households with a low, medium and high education.

b Data derived from the DNFCS 2012–2016 report. The nutrient constraints were calculated by taking the average intake in grams of macronutrients and food groups ±30%, except for energy (which was equal to the mean intake ± 5%). Nutrient targets for current diets vary for the four different households.

### 2.5 Healthy and current food baskets

Based on the commonly consumed food lists, four current and four healthy food baskets were constructed. These baskets contain food items and their corresponding weekly serving size constraints. Serving sizes were identified on the website of the National Institute for Health and Environment (RIVM, 2017). For example, a bowl of yoghurt is approximately 150 g, and the minimum serving of yoghurt is 75 g (i.e. half a portion per week) and the maximum serving is 2100 g (i.e. 14 servings of yoghurt per week). More servings were given to the most frequently consumed food items. Several meal plans were constructed by varying the serving size of foods found within the healthy and current food baskets while remaining within the nutrient constraints set for healthy and current diets. Furthermore, there is also at least half a serving size difference between different meal plans.

Both current as well as healthy food baskets contained food items in several food groups: fruit, vegetables, starchy vegetables, grains, dairy, protein foods, fats and oils, sauces (i.e. sauces, dressings, spreads and sugars), discretionary foods (i.e. confectionery, sweet biscuits, savoury snacks, cakes, pastries and puddings), beverages and alcohol. While takeaway foods may be included in the program, the current study did not include takeaway foods as their prices were not collected. The minimum serving size for each food item included in healthy and current diet baskets is 0.5, except for food items within the discretionary food group in the healthy diet basket where it was zero. As the discretionary food group was the only food group in the healthy diet basket that did not require at least a 0.5 serving per food item, most food items from the categories sauces and proteins were included in the category discretionary foods.

### 2.6 Food prices

A Dutch food price database was used to link food prices to the commonly consumed food items. A detailed description of the database can be found elsewhere (Mackenbach et al., 2019). Briefly, prices were collected in the summer of 2017. Researchers collected the retail prices for 902 food items commonly included in Dutch food frequency questionnaires (including alcohol). The lowest non-promotion price was included in the price database. Data was collected in two Dutch supermarket chains located in Amsterdam, the Netherlands. Prices were also collected from local food shops such as bakeries and butchers in Amsterdam. All prices in the food price database were adjusted for preparation and waste and were expressed in Euros (€) per 100g edible portion. While researchers are able to adjust the prices of products within the data files itself (e.g. Appendix A), the prices in the Dutch food price database were already adjusted for preparation and waste. As such, all waste factors were set to 1 in the input file. Prices of takeaway foods were not collected.

The food items within the price database were then linked to the commonly consumed food items in the Dutch food consumption survey. Of the n = 200 commonly consumed food items for the general population, n = 114 food items were directly linked using the NEVO code and n = 86 food items were indirectly linked. An indirect link included finding a comparable product with a known price and linking it to the product within the food consumption survey. For example, we used the price of wholemeal bread with NEVO code 246 and linked it to the food item coarse wholemeal bread with NEVO code 2782.

### 2.7 Affordability

According to the INFORMAS framework's ‘optimal’ approach to assess the cost, price differential and affordability of healthy diets (Lee et al., 2013), healthy diets are considered unaffordable if it costs more than 30% of household income. The Dutch Central Bureau of Statistics states that the average standardized net household income for the Dutch population in 2016 was €33,500/year (i.e. €1196/fortnight) (CBS, 2019). We could not find information on the standardized net household income by educational attainment. Compared to average Dutch individuals, Statista reported that the average gross annual salary in the Netherlands in 2021 was 27.8% higher for individuals with a high education, 10.5% lower for individuals with a medium education and 21.8% lower for individuals with a low education compared to the average population (Statista, 2021). Assuming this difference between educational levels is similar for net household income and has not changed in the last 5 years, we used this information to calculate the average standardized net household income by educational level (i.e. 935/fortnight, €1070/fortnight and €1528/fortnight for households with a low, medium and high educational level, respectively).

### 2.8 Analyses

Information on the DIETCOST program interface is reported elsewhere (Mackay et al., 2017; Vandevijvere et al., 2018). Briefly, using a list of commonly consumed foods with nutritional information, serving size and price data, the DIETCOST program constructs multiple meal plans that comply to the nutrient targets and constraints set for current and healthy diet scenarios. The DIETCOST program algorithm uses a random number generator to specify the starting meal plan and starting values in grams for each common food item. When a meal plan meets all targets and constraints, it is added to the results (Fig. 1). For each household member, the current and healthy diet scenarios were run 500,000 times. Iterations were increased with increments of 500,000 if the program did not arrive at an accurate estimate of the average cost of healthy and current diets (i.e. less than €1 difference between runs).

The variation in average cost and energy is a result of the multiple meal plans created by the DIETCOST program for each individual household member, which are then combined in order to calculate the average cost of healthy and current diets for the entire household. As the total number of possible combinations of household meal plans was too large to construct, a selection of combined bi-weekly meal plans for healthy and current household diets were made using bootstrapping. Bootstrapping was used to select a random number (n = 1000 through n = 10,000) of combined bi-weekly meal plans (seed set at 1234). The range and distribution of the cost of the fortnightly household meal plans and the contributions of each food group to the cost of diets was calculated. Percentage changes were calculated as (costhealthydiet–costcurrentdiet)/costcurrentdiet*100. Bootstrapping and descriptive statistics were conducted in RStudio version 4.0.3.

## 3 Results

On average, current meal plans required less iterations compared to healthy meal plans in order to produce a stable average cost (Table 2). Also, there was a large variety in number of potential meal plans constructed, ranging from 13 to 381.

**Table 2:** Average (SD) cost in the Netherlands and energy density of bi-weekly household healthy and current diets.

	Average household^a^		Low educated household		Medium educated household		High educated household	
	Healthy diets	Current diets	Healthy diets	Current diets	Healthy diets	Current diets	Healthy diets	Current diets
N iterations^b^	1M–4M	1M–1.5M	1M–4M	500,000–1M	2M–3M	1M	1M–5M	1M
N commonly consumed foods included	108	135	72	71	81	95	90	98
N individual meal plans for girls, boys, women and men	13 + 228+16 + 170	214 + 112+276 + 57	52 + 48+79 + 124	275 + 381+181 + 239	47 + 70+49 + 49	157 + 215+289 + 253	13 + 30+46 + 53	188 + 230+208 + 205
N bootstrap household meal plans	5000	5000	5000	5000	5000	5000	5000	5000
Average cost in € (SD)	260.8 (16.6)	210.6 (8.9)	200.7 (15.7)	183.2 (12.5)	240.3 (15.7)	190.3 (9.5)	269.4 (16.4)	197.9 (10.3)
Range of cost in €	214.9–321.1	179.8–245.1	153.6–272.7	148.4–229.7	182.1–300.6	158.6–224.9	214.5–319.9	165.2–244.5
Average Energy in mJ (range)	438.2 (415.5–450.3)	505.7 (493.1–526.6)	437.8 (414.4–446.7)	471.6 (459.4–495.5)	426.9 (405.8–444.7)	469.2 (458.3–495.7)	429.1 (409.8–445.9)	462.3 (446.4–486.6)

Abbreviations; M, million; SD, Standard Deviation; mJ, megajoule

a The average cost of healthy and current diets for the average Dutch household may not lie directly between that of low, medium and high educated households because 1) the commonly consumed food list for the average Dutch household is larger as it was based on a larger and more diverse population, and 2) the food items found in the commonly consumed food list of the average Dutch household mostly consists of foods consumed by medium and high educated individuals.

b The DIETCOST program constructs multiple meal plans that comply to the nutrient targets and constraints set for the current and healthy diet scenarios. This enables a comparison of the distribution of costs of current and healthy diets. For each individual household member, the current and healthy diet scenarios were first run at a minimum of 500,000 iterations to produce meal plans. If the program did not arrive at an accurate estimate of the average cost of healthy and current diets (i.e. less i.e. less than €1 difference between runs), then iterations were increased with increments of 500,000. Thus, the number of iterations and meal plans may vary between current and healthy diets and between households of varying educational levels.

None of the modelled current diets for either of the four household types (the average Dutch household and households with a low, medium and high education) met the dietary recommendations (Table 3 and Supplementary Table 1). This was largely because no household member met the maximum sodium intake guidelines and most household members did not meet the minimum intakes for fibre and fruit.

**Table 3:** Proportion of current diets meeting the guidelines for a healthy diet by each household member for the average household (individual meals for the current diet n = 214 girls, n = 112 boys, n = 276 women and n = 57 men).

Nutrient/food group	Healthy diet guideline	N girls (%)	N boys (%)	N women (%)	N men (%)
Fat in % energy	20–35	210 (98%)	105 (94%)	185 (67%)	49 (86%)
Saturated fat in % energy	0–10	89 (41%)	9 (8%)	0 (0%)	4 (7%)
Protein in % energy	15–25	17 (8%)	44 (39%)	152 (55%)	43 (75%)
Carbohydrates in % energy	45–65	295 (100%)	110 (99%)	269 (97%)	56 (99%)
Fibre in grams	18 + 36 + 30 + 35 (minimum)	15 (7%)	0 (0%)	0 (0%)	0 (0%)
Sodium in milligrams	962 + 2015 + 1493 + 1914 (maximum)	0 (0%)	0 (0%)	0 (0%)	0 (0%)
Red meat in grams	25 + 50 + 50 + 50 (maximum)	214 (100%)	112 (100%)	276 (100%)	36 (63%)
Dairy in servings	2 + 3 + 2 + 2 (minimum)	214 (100%)	112 (100%)	276 (100%)	57 (100%)
Vegetables in servings	1 + 2 + 2 + 2 (minimum)	214 (100%)	0 (0%)	202 (73%)	37 (65%)
Fruit in servings	2 (minimum)	158 (74%)	0 (0%)	0 (0%)	0 (0%)
All healthy diet guidelines		0 (0%)	0 (0%)	0 (0%)	0 (0%)

### 3.1 Cost and affordability of healthy and current diets

**Fig. 2:**
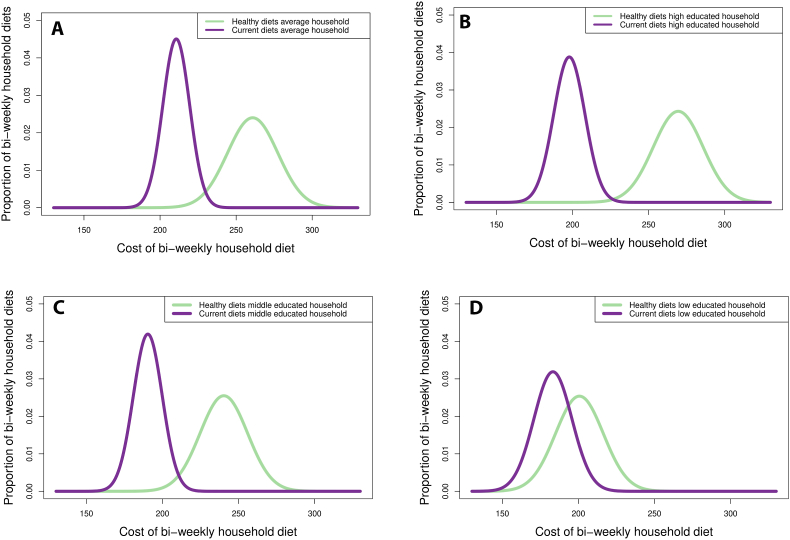
Distribution of the bi-weekly cost of healthy and current household diets separately for the average Dutch population (upper-left panel) and for the households with a low education (upper-right panel), medium education (lower-left panel) and high education (lower-right panel).

For all four types of households the average cost of healthy diets was higher than the average cost of current diets (Table 2). The average cost of healthy diets for the average Dutch household was €261/fortnight (SD 16.6), which was €50 (24%) costlier than the average cost of current household diets. Regarding the three different educational levels, the lowest healthy and current diet costs were found in households with a low education (e.g. current diets cost €183/fortnight (SD 12.5)), while the highest cost for both diets was found for the household with a high education (e.g. current diets cost 275 €198/fortnight (SD 10.3)). The average cost of healthy diets for the household with a high education was €69/fortnight higher than the average cost for the household with a low education. The percentage difference between the cost of healthy and current diets was 10% for the low, 26% for the medium and 36% for the high educated household. Also, 11% (561/5000), 0% (5/5000) and 0% (0/5000) of healthy diets were cheaper than the average cost of current diets for low, medium and high educated households, respectively. Fig. 2 represents the distribution of the bi-weekly cost of healthy and current household diets for all household types.

For all households, healthy diets were found to be affordable in terms of being below the aforementioned 30% threshold of household income. Namely, healthy diets cost the average Dutch household 22% of their disposable income (€260/€1196). For the households with a low, medium and high education this was 18%, 23% and 21%. According to Table 2 and Fig. 2, none of the healthy household diets would be considered unaffordable as the most expensive diet does not reach the 30% threshold. With regards to the affordability of current diets, this was 20%, 18% and 13% for households with a low, medium and high education, respectively.

### 3.2 Cost of food groups

**Fig. 3:**
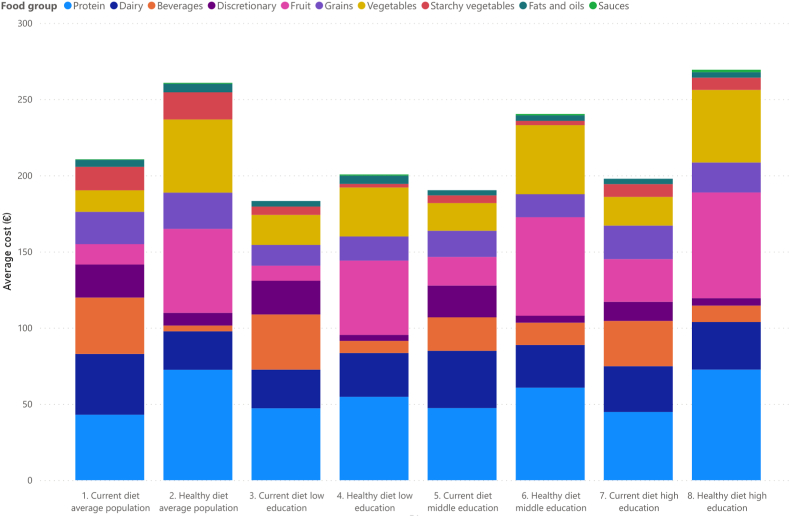
Contribution of food groups to the average cost of bi-weekly current and healthy diets for the different households.

Fig. 3 displays the contribution of food groups to the average cost of bi-weekly healthy and current diets for the four households (in Supplementary Table 2 the numeric values are found for all four types of households). All households would have to spend more money on protein, fruits and vegetables, and less money on discretionary foods and beverages if they were to follow the dietary recommendations.

**Fig. 4:**
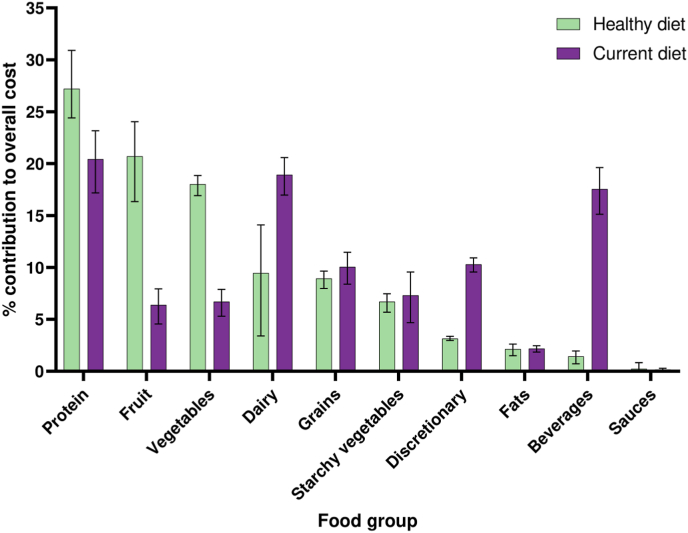
Percentage contribution of food groups to the overall cost of current and healthy diets of the average Dutch household with estimated 95% confidence interval.

Fig. 4 displays the relative contribution of food groups to the overall cost of healthy and current diets for the average household. Protein foods, fruits and vegetables have the largest overall contribution to the cost of a healthy diet; 27%, 21% and 18% of the cost of food can be contributed to protein foods, fruits and vegetables, respectively. The highest contributors to the cost of current diets were protein foods (20%), dairy (19%) and beverages (18%).

### 3.3 Diet scenarios

According to Table 4, the cost of current diets, including alcohol, slightly increased, while the cost of healthy diets remained relatively similar to a healthy diet without alcohol. When equating the amount of calories of the healthy diet to that of the current diet, the cost of healthy diets increases for all households (e.g. by €46/fortnight or 18%). The difference between healthy and current diets when equating their caloric content was €96/fortnight (or 46%), €42/fortnight (or 23%), €77/fortnight (or 41%) and €98/fortnight (or 50%) for the average Dutch households and the households with a low, medium and high education, respectively.

**Table 4:** Average (SD) cost in the Netherlands of bi-weekly healthy and current household diets for the total Dutch population and for low, medium and high educated households separately for the diet scenarios including alcohol and equal energy.

	Average household		Low educated household		Medium educated household		High educated household	
	Healthy diets	Current diets	Healthy diets	Current diets	Healthy diets	Current diets	Healthy diets	Current diets
Average cost of diet including alcohol in € (SD)	260.2	221.1	204.2	188.8	248.2	197.2	275.4	213.0
	(16.1)	(8.9)	(14.7)	(11.2)	(14.9)	(10.0)	(16.7)	(10.1)
Same energy in healthy and current diet without alcohol in € (SD)^a^	306.7	210.6	225.1	183.2	267.5	190.3	295.9	197.9
	(18.4)	(8.9)	(15.6)	(12.5)	(16.0)	(9.5)	(17.7)	(10.3)
Same energy in healthy and current diet including alcohol in € (SD)^b^	305.7	221.1	224.3	188.8	275.0	197.2	299.5	213.0
	(18.1)	(8.9)	(15.0)	(11.2)	(15.6)	(10.0)	(15.3)	(10.1)

Abbreviations; SD, Standard Deviation.

a The energy of current diets was used. As such, the cost of current diets were equal to the cost displayed in Table 2.

b The energy of current diets was used. As such, the cost of current diets including alcohol were equal to the cost displayed in the first column for current diets.

## 4 Discussions and conclusion

### 4.1 Discussion

This study modelled the cost of healthy and current household diets for the general Dutch population as well as for households with a low, medium and high education. For all four household types, the average cost of healthy diets is higher than the average cost of current diets. For the general Dutch household, healthy diets were on average €50/fortnight (i.e. €3.6/day or 24%) more expensive than current diets. This difference increased to €96/fortnight (or 46%) when equating the caloric content between healthy and current diets. The difference between healthy and current diets increased with increasing educational level. The higher cost of healthy diets was explained by the increased expenditure on protein foods, fruits and vegetables whereas individuals would have to spend less, i.e. save money, on discretionary foods and beverages.

The realization that an average Dutch household spends on average €260.9/fortnight (€18.6/day) on a healthy diet is somewhat higher compared to previous estimations by the Dutch National Institute for Family Finance Information showing that the minimum cost of food for a four-person Dutch household is €222.7/fortnight (€15.9/day) (NIBUD, 2020). However, the estimation by the Dutch National Institute is similar to the minimum cost of €214.9/fortnight (€15.5/day) for the average household, and is between the average cost of €200.7/fortnight (€14.3/day) and €240.3/fortnight (€17.1/day) for low and medium educated households. The comparatively high cost of the healthy diet may partly be explained by the larger variety of food items in the healthy food basket in our study. As no calculations for the cost of current diets were available, we could not compare our results to previous findings in the Netherlands.

Our study demonstrated that adhering to dietary recommendations is costlier than consuming current diets, which is in concordance with many (Jensen et al., 2015; Rao et al., 2013; Vandevijvere et al., 2018) but not all studies (Batis et al., 2021; Carlson & Frazão, 2012; Lee et al., 2020) in this field. Contrary to our findings, a recent Mexican study using the DIETCOST program found that current diets were more expensive than healthy diets (namely 40% or 29% when the comparison was isocaloric) (Batis et al., 2021), which is in line with another recent Australian study (Lee et al., 2020). Both Mexico and Australia have pricing policies in place (i.e. sugar sweetened beverages taxes and the exemption of GST on fruit and vegetables (Colchero et al., 2016; Veerman & Cobiac, 2013)) that may partly explain the different results. Our findings are in line with a previous DIETCOST study that found that for the New Zealand population, healthy diets were on average $27/fortnight (i.e. €1.1/day or 4%) more expensive than current diets (Vandevijvere et al., 2018). This finding is similar to a review that found that healthier food-based diet patterns were $20.7/fortnight (€1.2/day) more expensive than unhealthier diet patterns (Rao et al., 2013).

The current cost differential of 24% (or €3.6/day) is much larger compared to previous studies. This cost differential may partly be explained by the fact that the current study did not include takeaway meals, unlike these aforementioned studies (Lee et al., 2020; Vandevijvere et al., 2018). Indeed, a previous study showed that the average cost of current diets increased when including takeaways (Vandevijvere et al., 2018). Thus, if takeaways had been included in the current study, it is likely that the cost differential between healthy and current diets would be smaller. This cost differential is also in line with another European study; Danish researchers found that the healthy New Nordic diet was 16% more expensive than the current Danish diet (Jensen et al., 2015). Similarly, whereas we found that healthy diets were 46% costlier than current diets when equating the energy content, a study found that diets meeting six or more dietary recommendations in the United Kingdom were 29% costlier than diets with similar energy content meeting no recommendations (Jones et al., 2018). Prices of food and non-alcoholic beverages in the Netherlands are lower compared to other European countries such as Denmark and France (Eurostat, 2021). The relatively cheap price of food combined with the finding by the Dutch Central Bureau of Statistics suggesting that the price of healthier foods increased more than that of unhealthy foods (21% compared to 15%) (CBS, 2020), may explain the current study results. These contextual factors highlight the importance of conducting country-specific studies on the differential cost and affordability of healthy and current diets.

We further observed that the household with a low education had the lowest dietary costs and that the cost differential increased with increasing educational level (from 10% to 36%). In contrast to our findings, the previous Danish study found that the cost differential between current and healthy diets remained relatively stable for the lowest income class compared to the highest income class (Jensen et al., 2015). Households with low education cut costs when they switched to healthier diets, because of reduced intake of discretionary food. Households with higher education can lower their cost if they switch to healthier diets, like consuming more of cheaper but healthier diet items they currently do not consume (e.g. instead of salmon they could switch to lean fish). Nevertheless, despite the increasing cost differential found in this study, healthy diets were approximately equally affordable across the different households (costing around 20% of their household income). This does not imply that for the Dutch, the cost of food is not hindering healthy dietary choices; studies conducted in the Netherlands show that the relatively high price of healthy foods compared to unhealthy foods can still be an important barrier to adopting healthy diets, especially for individuals with a low SEP (Dijkstra et al., 2014; Steenhuis et al., 2011).

Strengths of the study include the use of the DIETCOST program allowing researchers to generate multiple shopping lists for bi-weekly meal plans that meet the targets and constraints for both current and healthy diets (Vandevijvere et al., 2018). Unlike studies to date that have compared the cost of one healthy and one current diet, DIETCOST allows for the cost of many fortnightly household diets to be generated. Additionally, common foods, nutrient and food group targets for households with different educational levels allowed for the modelling for these specific populations. A limitation is that the cost of healthy and current diets does not reflect the actual expenditure as price data regarding the cheapest available product was used instead of the price of the actual consumed product. This conservative approach has likely led to an underestimation in the cost differences between households with a low, medium and high education. Given their limited food budget, individuals with a low SEP may be more likely to purchase the cheapest version of a food item compared to individuals with a higher SEP (e.g. home-brand versus name-brand food items and food items on promotion) (Zorbas et al., 2020). Another limitation is that we did not collect data on the price of takeaway meals, which has likely led to an underestimation of the cost of current diets given that takeaway meals are generally unhealthy, which in turn could lead to an overestimation of the difference in cost of healthy and current diets. A third limitation is that while the DIETCOST program allows for variation in food prices by shop, region and/or over time, this was not included in the current study. Indeed, recent evidence shows that the price of foods increased by 18% from 2011 to 2021 and that this price increase differed by food group (CBS, 2020). A last limitation is the use of educational level as a proxy for SEP as income is likely more related to dietary expenditure compared to educational level. Unfortunately, income data for all participants of the DNFCS 2012–2016 was not available.

Future research should consider the actual food expenditure, including details on buying promotional items, going to different supermarkets etc., differentiated by SEP. Furthermore, comprehensive monitoring of the price of food and beverages can inform policy action. For example, as evidence suggests that the price of healthier foods increased more than that of unhealthier foods (CBS, 2020), the cost difference between healthy and current diets may have actually increased since the data collection of the current study. Since the cost differential of healthy versus current diets for the households with a low education is only around 10%, it would be of interest to investigate which other factors hamper households with a lower SEP to adopt healthier diets. Such factors may include both diet-related factors such as cooking skills and nutrition knowledge as well as more upstream factors related to social causes (e.g. inadequate and temporary housing, discrimination and stigma) (Kumanyika, 2019).

The current study findings indicate that healthy diets are more costly than less healthy diets, which may partly hinder the adoption of healthier diets. Indeed, previous studies found that the higher cost of healthier foods partly explains socioeconomic differences in diet (Monsivais et al., 2012; Pechey & Monsivais, 2016), also in the Netherlands (Hoenink et al., 2020). As such, it may be important to bridge the gap in cost between healthy and less healthy diets. Evaluations of policy interventions indicate that sugar sweetened beverages taxes are effective in decreasing beverage purchases (Teng et al., 2019). Other policy interventions may include fruit and vegetable incentive programs for increasing fruit and vegetable purchases and consumption among vulnerable populations (Engel & Ruder, 2020).

### 4.2 Conclusion

Although healthy diets were found to be affordable in terms of disposable income, healthy diets were on average 24% more expensive than current diets in the Netherlands. This increased cost was mainly due to the higher required expenditure on protein foods, fruits and vegetables. Both the cost of and cost differential between healthy and current diets was lowest for the household with a low education and highest for the household with a high education if one assumes that households would only switch to other commonly consumed foods, and not to rarely consumed foods. Policy interventions may want to bridge the gap between the cost of healthy and less healthy (current) diets to improve population diets.

## Author contributions

Jody C Hoenink: Conceptualization, Formal analysis, Data Curation, Visualization, Writing - Original Draft, Stefanie Vandevijvere: Methodology, Software, Resource, Writing - Review & Editing, Wilma Waterlander: Conceptualization, Supervision, Writing - Review & Editing, Joline WJ Beulens: Conceptualization, Supervision, Writing - Review & Editing, Funding acquisition, Joreintje D Mackenbach: Conceptualization, Supervision, Writing - Review & Editing.

## Ethical standard disclosure

All data were obtained from publicly available sources and did not involve human participants.

## Financial support

JCH and JDM are funded by the Netherlands Heart Foundation and the Netherlands Organization for Health Research and Development (10.13039/501100001826ZonMw) through the Supreme Nudge (CVON 2016–04) project.

## Declaration of competing interest

The authors have no competing interests.

## Data Availability

Data is publicly available.
