# Supplementary material for: The cost of healthy versus current diets in the Netherlands for households with a low, middle and high education
Source: SSM Popul Health. 2022 Nov 25;20:101296. doi: 10.1016/j.ssmph.2022.101296 (PMC9712664; doi:10.1016/j.ssmph.2022.101296)
Supplement: Multimedia component 2 [file mmc2.docx]

# Supplementary Material

**Supplementary Table 1.** Proportion of current diets meeting the guidelines for a healthy diet by each household member for low, middle and high educated households

| **Nutrient/food group** | **Healthy diet guideline** | **N girls (%)** | | | **N boys (%)** | | | **N woman (%)** | | | **N man (%)** | | |
| --- | --- | --- | --- | --- | --- | --- | --- | --- | --- | --- | --- | --- | --- |
|  |  | **Educational level** | | | | | | | | | | | |
|  |  | **Low** | **Middle** | **High** | **Low** | **Middle** | **High** | **Low** | **Middle** | **High** | **Low** | **Middle** | **High** |
| Fat in % energy | 20-35 | 270 (98%) | 124 (79%) | 188 (100%) | 368 (97%) | 213 (99%) | 229 (100%) | 131 (72%) | 244 (84%) | 173 (83%) | 198 (85%) | 188 (74%) | 154 (75%) |
| Saturated fat in % energy | 0-10 | 118 (43%) | 48  (31%) | 55  (29%) | 129 (34%) | 45  (21%) | 66  (29%) | 34  (19%) | 25  (9%) | 18  (9%) | 69  (29%) | 18  (7%) | 15  (7%) |
| Protein in % energy | 15-25 | 57  (21%) | 48  (31%) | 17  (9%) | 85  (22%) | 85  (40%) | 13  (6%) | 67  (37%) | 179 (62%) | 36  (17%) | 92  (39%) | 195 (77%) | 65  (32%) |
| Carbohydrates in % energy | 45-65 | 275 (100%) | 157 (100%) | 188 (100%) | 381 (100%) | 215 (100%) | 228 (100%) | 171 (94%) | 275 (95%) | 199 (96%) | 228 (97%) | 215 (85%) | 192 (94%) |
| Fibre in grams | 18 + 36 + 30 + 35 (minimum) | 37  (13%) | 10  (6%) | 27  (14%) | 0  (0%) | 0  (0%) | 0  (0%) | 0  (0%) | 0  (0%) | 0  (0%) | 0  (0%) | 0  (0%) | 0  (0%) |
| Sodium in milligrams | 962 + 2015 + 1493 + 1914 (maximum) | 0  (0%) | 0  (0%) | 0  (0%) | 50  (13%) | 19  (9%) | 141  (61%) | 0  (0%) | 0  (0%) | 0  (0%) | 0  (0%) | 0  (0%) | 0  (0%) |
| Red meat in grams | 25 + 50 + 50 + 50 (maximum) | 0  (0%) | 0  (0%) | 79  (42%) | 280 (73%) | 126 (59%) | 230 (100%) | 84  (46%) | 289 (100%) | 208 (100%) | 22  (9%) | 32  (13%) | 205 (100%) |
| Dairy in servings | 2 + 3 + 2 + 2 (minimum) | 195 (71%) | 132 (84%) | 138 (73%) | 125 (33%) | 128 (59%) | 48  (21%) | 164 (91%) | 274 (95%) | 180 (87%) | 234 (100%) | 253 (100%) | 205 (100%) |
| Vegetables in servings | 1 + 2 + 2 + 2 (minimum) | 275 (100%) | 157 (100%) | 188 (100%) | 0  (0%) | 0  (0%) | 0  (0%) | 106 (59%) | 185 (64%) | 193 (93%) | 77  (33%) | 209 (83%) | 193 (94%) |
| Fruit in servings | 2 (minimum) | 0  (0%) | 0  (0%) | 0  (0%) | 0  (0%) | 0  (0%) | 0  (0%) | 0  (0%) | 0  (0%) | 31  (15%) | 0  (0%) | 0  (0%) | 0  (0%) |
| All healthy diet guidelines |  | 0  (0%) | 0  (0%) | 0  (0%) | 0  (0%) | 0  (0%) | 0  (0%) | 0  (0%) | 0  (0%) | 0  (0%) | 0  (0%) | 0  (0%) | 0  (0%) |

**Supplementary Table 2**. Average household cost by food groups

|  | Overall population | | Low education | | Middle education | | High education | |  |
| --- | --- | --- | --- | --- | --- | --- | --- | --- | --- |
|  | Healthy diets | Current diets | Healthy diets | Current diets | Healthy diets | Current diets | Healthy diets | Current diets | |
| Fruit | 55.2 (8.4) | 13.5 (2.4) | 48.8 (7.3) | 9.9 (1.1) | 64.7 (9.2) | 18.8 (3.5) | 69.5 (9.3) | 28.0 (5.1) | |
| Vegetables | 48.0 (8.9) | 14.1 (2.0) | 32.1 (8.2) | 19.7 (4.7) | 45.3 (9.0) | 18.1 (3.4) | 47.6 (8.3) | 18.8 (4.1) | |
| Starchy vegetables | 17.8 (4.6) | 15.4 (3.3) | 2.4 (0.8) | 5.5 (0.6) | 2.9 (0.9) | 5.1 (0.5) | 8.1 (2.3) | 8.4 (2.2) | |
| Grains | 23.8 (2.4) | 21.2 (2.5) | 15.9 (1.6) | 13.7 (1.5) | 15.0 (1.7) | 17.3 (2.3) | 19.7 (2.4) | 22.1 (2.7) | |
| Dairy | 25.2 (2.7) | 39.9 (3.6) | 28.7 (3.5) | 25.4 (2.6) | 27.9 (2.8) | 37.6 (4.7) | 31.2 (3.1) | 30.0 (3.2) | |
| Protein | 72.5 (8.9) | 43.0 (5.0) | 54.8 (10.6) | 47.2 (9.2) | 60.8 (8.5) | 47.4 (6.4) | 72.6 (9.4) | 44.8 (5.6) | |
| Fats and oils | 5.7 (0.6) | 4.6 (0.5) | 5.4 (0.6) | 3.6 (0.5) | 3.6 (0.5) | 3.4 (0.4) | 3.6 (0.5) | 3.5 (0.5) | |
| Discretionary foods | 8.4 (1.1) | 21.7 (1.6) | 3.9 (0.8) | 22.1 (2.0) | 4.6 (0.9) | 20.8 (1.8) | 4.8 (1.1) | 12.5 (0.8) | |
| Sauces | 0.6 (0.3) | 0.0 (0.0) | 0.8 (0.4) | 0.0 (0.0) | 0.89 (0.4) | 0.0 (0.0) | 1.6 (0.6) | 0.0 (0.0) | |
| Beverages | 3.8 (1.0) | 37.0 (4.0) | 7.9 (1.3) | 36.2 (6.1) | 14.7 (2.9) | 21.9 (4.0) | 10.8 (2.8) | 29.7 (4.0) | |
| Total | 266.3 (18.1) | 210.6 (8.9) | 200.7 (15.7) | 183.2 (12.5) | 240.3 (15.7) | 190.3 (9.5) | 269.4 (16.4) | 197.9 (10.3) | |
